# Insights into the Composition and Structural Chemistry of Gallium(I) Triflate

**DOI:** 10.1002/anie.202010837

**Published:** 2020-11-23

**Authors:** Josef T. Boronski, Matthew P. Stevens, Bono van IJzendoorn, Adrian C. Whitwood, John M. Slattery

**Affiliations:** ^1^ Department of Chemistry The University of York Heslington York YO10 5DD UK

**Keywords:** coordination chemistry, density functional calculations, low-valent gallium, main-group chemistry, X-ray crystallography

## Abstract

“GaOTf” is a simple, convenient source of low‐valent gallium for synthetic chemistry and catalysis. However, little is currently known about its composition or reactivity. In this work, ^71^Ga NMR spectroscopy shows the presence of [Ga(arene)_*n*_]^+^ salts on oxidation of Ga metal with AgOTf in arene solvents. However, a more complex picture of speciation is uncovered by X‐ray diffraction studies. In all cases, mixed‐valence compounds containing Ga‐arene and Ga‐OTf coordination motifs, in addition to an unusual “naked” [Ga]^+^ ion, are found. Addition of 18‐crown‐6 allows for the isolation of a discrete Ga^I^ crown complex. Evidence of a potential intermediate in the formation of “GaOTf” has been isolated in the form of the bimetallic silver(I)/gallium(I) cluster anion [Ag_4_{Ga(OTf)_3_}_4_(μ‐Ga)_6_(OTf)_4_]^2−^.

## Graphical abstract

Gallium(I) triflate is a promising new source of low‐valent gallium for synthesis and catalysis. However, little is known about its composition and structural chemistry. Here it is demonstrated that speciation is quite complex in this system, with compounds containing Ga in multiple oxidation states being isolated from arene solutions.

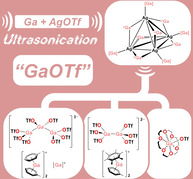

Gallium occupies a fascinating position in Group 13. Much of its chemistry is dominated by the +3 oxidation state, like the lighter elements, but it also displays an extensive range of low oxidation state compounds. There have been a number of breakthroughs in the development of starting materials for low‐valent gallium chemistry, each of which has advantages and disadvantages. True Ga^I^ halides are thermodynamically unstable, and while they can be trapped and utilized, this requires specialist equipment.^[1]^ Ga_2_Cl_4_ (formulated as Ga^+^[GaX_4_]^−^) has been known for many years, and has an extensive coordination chemistry with arene ligands,^[2]^ but often undergoes dis‐ or comproportionation on reaction with other species. “GaI” can be an excellent source of Ga^I^, but like Ga_2_Cl_4_ also has a tendency towards unexpected behavior.^[3]^ [Ga_2_C_5_Me_5_][B(Ar^F^)_4_] salts are capable of delivering Ga^+^ ions as ligands to suitable transition‐metal complexes.^[4]^ [Ga(arene)_*n*_]^+^[Al(OC(CF_3_)_3_)_4_]^−^ (*n*=2, 3) salts are well‐behaved sources of Ga^I^, but these anions are still not as widely used as simple weakly coordinating anions (WCAs) such as triflate.^[5]^ More recently, Ga^I^ species have been accessed via reductive elimination of H_2_ from Ga^III^ dihydrides.^[6]^ Furthermore, a family of Ga‐based carbenoids, with differing charges and ring sizes, have emerged as important building blocks in low‐valent Ga chemistry and have been used as ligands in coordination chemistry.^[7]^

There are a growing number of reports of Ga^I^ involvement in catalysis and the development of new, more accessible, well‐behaved low‐valent gallium sources is particularly important to allow this area to grow. [Ga(arene)_*n*_]^+^ salts catalyze the polymerization of alkenes,[^5e^, ^5f^, ^6^] and have been shown to be active catalysts for cycloisomerization, transfer hydrogenation, and reductive hydroarylation reactions.^[9]^ There is also evidence for Ga^I^ involvement in important mechanistic steps that underpin many catalytic reactions, for example, reductive elimination, suggesting exciting possibilities for the Ga^I^/Ga^III^ redox couple in other catalytic reactions.[^3c^, ^6^] In related work, the use of Ga^0^ in synthesis and materials chemistry has received attention.^[10]^

Schneider et al. recently reported the preparation of a new low‐valent gallium reagent “GaOTf” (OTf=[O_3_SCF_3_]^−^) using a similar route to the preparation of [Ga(arene)_*n*_]^+^[Al(OC(CF_3_)_3_)_4_]^−^ salts,^[5a]^ where gallium metal is oxidized by AgOTf in arene solvents, or dioxane in the presence of 18‐crown‐6.^[8]^ “GaOTf” solutions were then used as ambiphilic catalysts for C−C bond forming reactions (Figure 1). Control experiments with Ga^III^ salts, Ag^0^ or other metal triflates showed that the presence of low oxidation‐state Ga was important for catalytic activity. ^71^Ga NMR spectroscopy suggested the formation of a [Ga(18‐crown‐6)][OTf].(dioxane)_*n*_ complex under catalytic conditions and other stoichiometric studies allowed a catalytic cycle involving Ga^I^‐alkoxide and ‐allyl intermediates to be proposed. However, speciation in “GaOTf”, particularly in arene solvents, was not explored in detail. Given the potential wider uses of “GaOTf” as one of the most easily accessible sources of Ga^I^ yet reported, herein we explore the composition and structural chemistry of the “GaOTf” system in detail for the first time.

**Figure 1:**
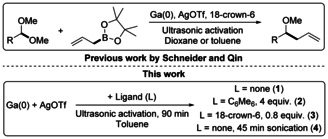
Low‐valent‐Ga catalyzed reaction between acetals, ketals, or aminals and allyl boronic esters reported by Schneider (top)^[8]^ and this work (below).

Reaction of AgOTf and Ga metal in toluene under ultrasonic activation (see Figure 1 for summary) led to the immediate formation of a pale‐yellow solution and after ca. 1.5 hours of ultrasonication a dense black precipitate. After filtration, ^71^Ga NMR spectroscopy showed a single, relatively sharp peak at −692 ppm, which is characteristic of a gallium(I) arene complex.^[2]^ Storage of the solution overnight at −20 °C led to the formation of pale brown crystals, which X‐ray crystallography revealed to contain [Ga][Ga(C_6_H_5_Me)_2_]_2_[Ga_3_(OTf)_8_] (**1**; Figure 2).

**Figure 2:**
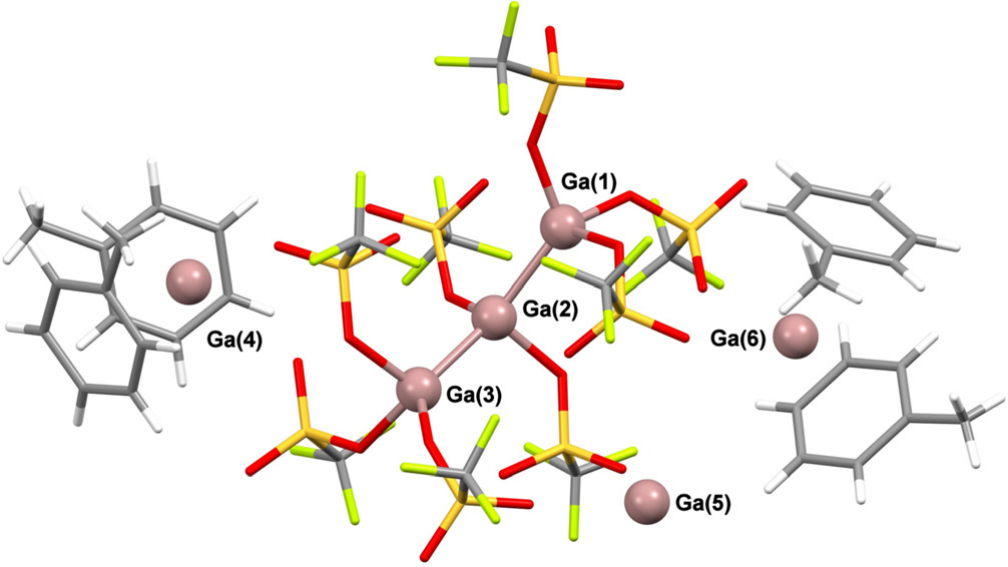
Crystal structure of [Ga][Ga(C_6_H_5_Me)_2_]_2_[Ga_3_(OTf)_8_] (**1**).^[30]^ Monoclinic, *P*2_1_/*c*, 110 K, *R*1=0.0784, wR_2_(all)=0.2303. Some toluene molecules and one [OTf]^−^ are disordered over two positions. Disordered parts are omitted for clarity. Selected bond lengths [Å] and angles [°]: Ga1–Ga2 2.379(1), Ga2–Ga3 2.377(1), Ga4–C_6_(cent) 2.837(8) and 2.861(4), Ga6–C_6_(cent) 2.809(12) and 2.831(5); Ga1‐Ga2‐Ga3 140.31(6), C_6_(cent)‐Ga4‐C_6_(cent) 121.30(18), C_6_(cent)‐Ga4‐C_6_(cent) 126.95(30).

As anticipated, and consistent with the ^71^Ga NMR spectrum, **1** contains two bent‐sandwich gallium(I) toluene cations, [Ga(C_6_H_5_Me)_2_]^+^, which have very similar parameters to the bis‐benzene complex reported by Schmidbaur [Ga(C_6_H_6_)_2_][GaCl_4_] (av. Ga‐C_6_ centroid in **1**=2.835 vs. 2.844 Å, av. C_6_(cent)‐Ga‐C_6_(cent) in **1**=124 vs. 124°).^[11]^ In addition, an unusual “naked” [Ga]^+^ ion, which is stabilized by Ga⋅⋅⋅O and Ga⋅⋅⋅F contacts with the anions, rather than arene coordination, is found in the asymmetric unit.

The counterions in **1** are not simple [OTf]^−^ ions, but the previously unknown trianion [Ga_3_(OTf)_8_]^3−^. This is composed of a catenated [Ga_3_]^5+^ core, coordinated by eight [OTf]^−^ and is related to the Ga_3_X_5_L_3_ motif identified by Schnöckel in [Ga_3_I_5_(PEt_3_)_3_],^[12]^ the monoanion [Ga_3_(OTf)_6_(GaCp*)_2_]^−^ reported by Linti^[13]^ and Baines’ [Ga_3_Cl_4_(crypt‐222)]^+^ cation.^[14]^ Indeed, these species can all be viewed as Ga_3_X_5_L_3_‐type compounds, where X=I, Cl, or OTf and L is either a neutral (PEt_3_, GaCp* or crypt‐222) or anionic ([OTf]^−^) ligand. In Baines’ system one X^−^ has been lost and the [Ga_3_]^5+^ core forms an additional bond to the cryptand to compensate for this. Comparing the structural parameters of [Ga_3_(OTf)_8_]^3−^ with other Ga_3_X_5_L_3_‐type compounds shows that the average Ga−Ga bond lengths are shorter in [Ga_3_(OTf)_8_]^3−^ (2.374 Å, compared to 2.456 Å in [Ga_3_I_5_(PEt_3_)_3_], 2.426 Å in [Ga_3_(OTf)_6_(GaCp*)_2_]^−^ and 2.416 Å in [Ga_3_Cl_4_(crypt‐222)]^+^). These species are all mixed‐valence Ga compounds and different oxidation state models have been proposed. In [Ga_3_(OTf)_8_]^3−^ a Ga^II^‐Ga^I^‐Ga^II^ model, in analogy to [Ga_3_I_5_(PEt_3_)_3_], would seem appropriate. However, recent XAS studies suggest that assignment of formal oxidation states in such species is challenging and a Ga^I^‐Ga^III^‐Ga^I^ model is also feasible.^[14b]^ Other examples of catenated Ga and In species, some with quite extended structures, have been reported and are of note.[^7e^, ^12^, ^13^, ^15^]

In an attempt to prevent disproportionation and the formation of higher oxidation state species, “GaOTf” was prepared as described above, but with the addition of four equivalents of hexamethylbenzene, as a more π‐basic arene.^[16]^ The ^71^Ga NMR spectrum after ultrasonication and filtration featured a single peak at −707 ppm. Colorless crystals were grown by storage of the solution at −20 °C and X‐ray diffraction studies showed these to have the composition [Ga(C_6_H_5_Me)(C_6_Me_6_)]_2_[Ga_2_(OTf)_6_] (**2**; Figure 3).

**Figure 3:**
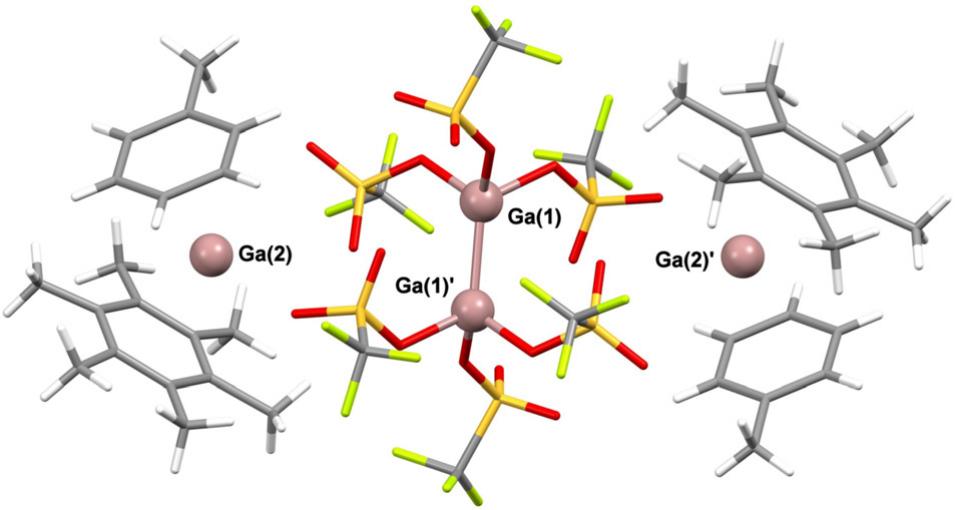
Crystal structure of [Ga(C_6_H_5_Me)(C_6_Me_6_)]_2_[Ga_2_(OTf)_6_] (**2**).^[30]^ Monoclinic, *P*2_1_/*n*, 110 K, *R*1=0.0861, wR_2_(all)=0.2444. The asymmetric unit contains half of the molecular formula, with the other half related to this by a center of inversion. A toluene molecule in the lattice, disordered over two positions, is omitted for clarity. Selected bond lengths [Å] and angles [°]: Ga1–Ga1′ 2.355(6), Ga2–C_6_Me_6_(cent) 2.525(9), Ga2–C_6_H_5_Me(cent) 2.813(11); C_6_(cent)‐Ga2‐C_6_(cent) 134.97(30).

The [Ga(C_6_H_5_Me)(C_6_Me_6_)]^+^ units in **2** are rare examples of mixed bis(arene) gallium(I) complexes.^[16]^ The Ga–(C_6_Me_6_) C_6_ centroid distance is one of the shortest known, at 2.525 Å.[^2^, ^17^] This is 0.288 Å shorter than the Ga–(C_6_H_5_Me) C_6_ centroid distance (2.813 Å) in **2**, suggesting a significantly enhanced interaction between Ga and the more π‐basic arene. Shorter Ga–arene distances have been observed in the mono‐arene salt [Ga(C_6_Me_6_)][Al(OC(CF_3_)_3_)_4_] (2.262 Å), but are otherwise rare.[^17a^, ^18^] The effects of the steric bulk of the C_6_Me_6_ ligand are clearly evident in the arene interplanar angles in **2**, which are considerably smaller (av. 43°) than those in **1** (av. 56°). Bent‐sandwich gallium(I) complexes of bulky arenes have previously been seen to display small interplanar angles, as they are forced into a more linear geometry in order to reduce the steric interaction between the two coordinating arenes.^[16]^

As in **1**, the counteranion in **2** is not a simple [OTf]^−^, but rather the Ga^II^ dianion [Ga_2_(OTf)_6_]^2−^.^[19]^ While the formal oxidation states of Ga in this anion are different to those in [Ga_3_(OTf)_8_]^3−^ their structural parameters are similar, with average Ga−Ga bond lengths of 2.378 Å in **1** and a Ga−Ga distance of 2.355 Å in **2**. Related, halide‐containing anions such as [Ga_2_I_6_]^2−^, which is thought to be a major component of Green's “GaI”,^[3]^ have also been seen in similar systems. Other Ga^II^ compounds that include Ga−Ga bonds, as here, have been known for some time.[^12^, ^20^] More recently, monometallic Ga^II^ radicals have also been isolated.^[21]^ The [Ga_2_(OTf)_6_]^2−^ and [Ga_3_(OTf)_8_]^3−^ polyanions seen here are presumably formed via partial disproportionation of Ga^I^ salts formed after oxidation of Ga by Ag^+^. This suggests that [OTf]^−^ is not a suitable anion for stabilizing Ga^I^ arene complexes. The larger, highly fluorinated polyanionic species can be viewed as in situ generated WCAs, which are less nucleophilic than [OTf]^−^ and as such are better able to stabilize [Ga(arene)_*n*_]^+^ ions, in analogy to the large [Al(OC(CF_3_)_3_)_4_]^−^ WCA.

It can now be seen that there are several isolable low‐valent‐gallium‐containing species, both cationic and anionic, within “GaOTf” solutions. The composition of the crystalline material is only a snapshot of some of the potential species present in solution. What is clear, however, is that “GaOTf” is more complex than it might initially appear and multiple Ga coordination environments and oxidation states are present. This realization is very important for the interpretation of catalytic studies involving “GaOTf”, as the catalytically active species could involve several possible Ga oxidation states.

Schneider et al. showed that addition of 18‐crown‐6 to “GaOTf” improved the yields in their catalytic C−C bond forming reactions.^[8]^ To investigate the potential for low‐valent Ga‐crown complex formation in this system, “GaOTf” solutions were prepared in toluene, as described above, filtered and the filtrate added to a solution containing 18‐crown‐6. In suitably concentrated solutions, this led to the spontaneous formation of colorless, block‐like crystals. This was encouraged by low temperature at the time of filtration. X‐ray diffraction studies revealed these to contain [Ga(18‐crown‐6)(OTf)] (**3**; Figure 4).

**Figure 4:**
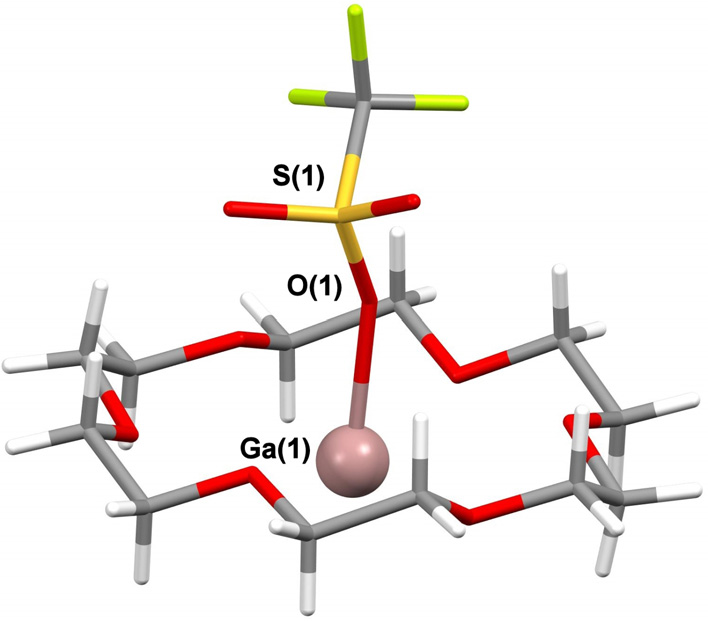
Crystal structure of [Ga(18‐crown‐6)(OTf)] (**3**).^[30]^ Monoclinic, *P*2_1_/*n*, 110 K, *R*1=0.0214, wR_2_(all)=0.0564. Selected bond lengths [Å] and angles [°]: Ga1–O1 2.137(1); Ga1‐O1‐S1 149.71(8).

Compound **3** is only the second structurally authenticated Ga^I^‐18‐crown‐6 complex, the first being [Ga(18‐crown‐6)(C_6_H_5_F)_2_][Al(OC(CF_3_)_3_)_4_].^[5b]^ However, these complexes differ significantly, as **3** features tight ion pairing, whereas [Ga(18‐crown‐6)(C_6_H_5_F)_2_][Al(OC(CF_3_)_3_)_4_] displays no contacts between the gallium cation and the more weakly coordinating alkoxyaluminate anion. Instead, weak Ga‐C_6_H_5_F interactions above and below the plane of the crown complete the coordination sphere around Ga. Coordination of [OTf]^−^ to Ga in **3** appears to lead to a weakening of the Ga–crown interactions, with average Ga–O(crown) distances of 2.840 and 2.800 Å for **3** and [Ga(18‐crown‐6)(C_6_H_5_F)_2_][Al(OC(CF_3_)_3_)_4_] respectively. Complex **3** can be compared to the isostructural indium salt [In(18‐crown‐6)(OTf)].^[22]^ In this case, the average In−O(crown) distance is longer (2.874 Å) than in **3**, suggesting that the larger In^+^ ion sits slightly above the crown, compared to Ga. The Ga‐OTf distance in **3** (2.137 Å) is 0.233 Å shorter than the In−OTf distance in [In(18‐crown‐6)(OTf)], closely mirroring the difference between the ionic radii of gallium(I) and indium(I) (0.23 Å).^[23]^ A related [Ga(12‐crown‐4)]^+^ salt has also been reported in a recent PhD thesis and coordination of Ga^+^ by the tridentate PMDETA ligand, somewhat related to crown ethers, is also noted for comparison.^[24]^

Unlike in **1** and **2**, the presence of 18‐crown‐6 in **3** appears to facilitate the formation of a Ga^I^ salt with a discrete [OTf]^−^ anion, rather than a complex Ga‐containing polyanion. This may be because the crown offers additional stability to Ga^I^ towards disproportionation, which could explain its positive impact in Schneider's catalysis.^[8]^ Indeed, in our hands **3** is considerably more stable than solutions from which **1** or **2** are isolated, which readily disproportionate over time or on removal of the solvent.

To explore the electronic structure of **3**, DFT studies were performed (see the Supporting Information for details). The optimized structure of **3** (at the (RI‐)BP86/SV(P) level) is shown in Figure 5. Comparisons between **3** and other gallium(I) species, such as GaCp*, can be drawn from analysis of the DFT data.^[4f]^ Natural bond orbital (NBO) calculations (at the (RI‐)PBE0/def2‐TZVPP//(RI‐)BP86/SV(P) level) suggest that there is a small amount of sp character to the Ga^I^ lone pair (3 % p character), which gives some directionality, as shown in Figure 5. This is very similar to the hybridization in GaCp* (4 % p character), which has an established coordination chemistry as a ligand to other metals, and suggests that **3** may prove to be an interesting new low‐valent Ga‐based ligand, an area under investigation in our laboratory.

**Figure 5:**
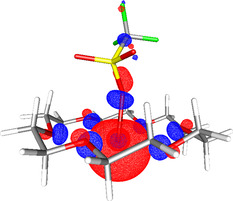
Optimized structure of **3** showing the HOMO (−6.24 eV, at the (RI‐)PBE0/def2‐TZVPP//(RI‐)BP86/SV(P) level).

To explore speciation during the formation of “GaOTf” at shorter time periods, a toluene solution of AgOTf and a bead of Ga was sonicated for less than one hour. Filtration produced an orange solution, from which block‐like orange crystals were formed after storage at −20 °C for four days. X‐ray diffraction studies revealed these to be salt (**4**), which is composed of two [Ga(C_6_H_5_Me)_2_]^+^ ions, the novel and unusual Ga‐Ag cluster ion [Ag_4_{Ga(OTf)_3_}_4_(μ‐Ga)_6_(OTf)_4_]^2−^ (Figure 6) and a toluene molecule of crystallization. In light of the presence of both Ag^I^ and low‐valent Ga species in **4**, we speculate that this may represent an intermediate species present at short reaction times during the formation of **1** or related systems. It is also a compound of significant fundamental interest and will be discussed below.

**Figure 6:**
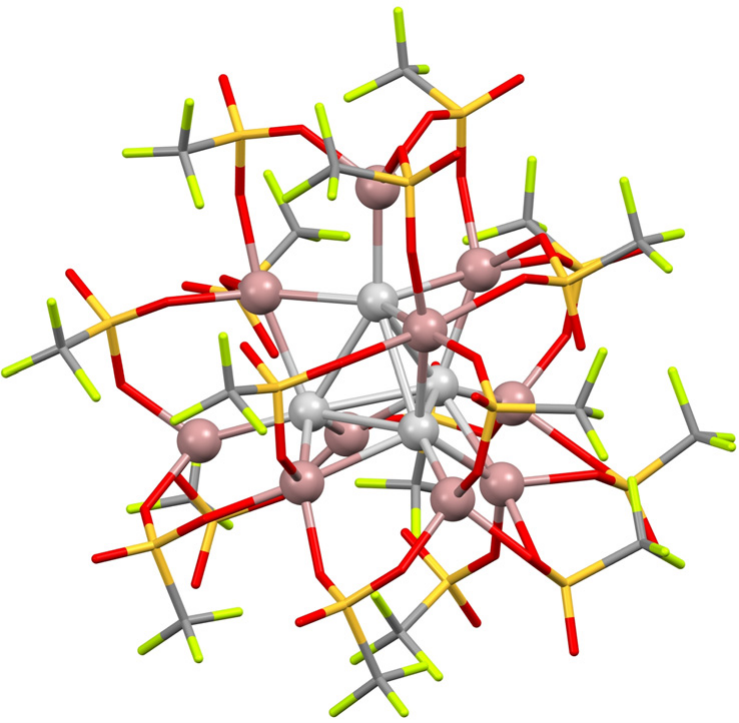
Molecular structure of the cluster anion from [Ga(C_6_H_5_Me)_2_]_2_[Ag_4_{Ga(OTf)_3_}_4_(μ‐Ga)_6_(OTf)_4_].C_6_H_5_Me (**4**).^[30]^ Monoclinic, *C*2/*c*, 110 K, *R*1=0.0625, wR_2_(all)=0.1828. Selected bond lengths [Å]: av. Ag–Ag 2.871, av. Ag–Ga_(vertex)_ 2.468, av. Ag–Ga_(edge)_ 2.631.

Although the overall XRD data quality for **4** are relatively poor, due to extensive disorder in the [Ga(C_6_H_5_Me)_2_]^+^ ions and toluene of crystallization, they are sufficient to confirm the composition of the salt and some discussion of the cluster anion, which is quite well resolved, is reasonable. The [Ag_4_{Ga(OTf)_3_}_4_(μ‐Ga)_6_(OTf)_4_]^2−^ anion in **4** can be viewed as being formed from a tetrahedral [Ag_4_]^4+^ core, coordinated by four [Ga(OTf)_3_]^2−^ ligands: one at each vertex. Bridging Ga^+^ ions coordinate each of the six edges of the tetrahedral [Ag_4_]^4+^ core, forming contacts with oxygen atoms from the [Ga(OTf)_3_]^2−^ units. The bridging Ga^+^ are further coordinated by the remaining four triflate ions, which sit roughly above each face of the tetrahedral [Ag_4_]^4+^ core. A simplified illustration is shown in Figure 7.

**Figure 7:**
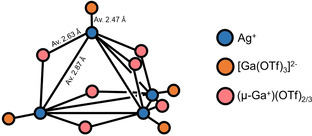
Simplified structure for [Ag_4_{Ga(OTf)_3_}_4_(μ‐Ga)_6_(OTf)_4_]^2−^.

Although uncommon, clusters based around a tetrahedral [Ag_4_]^4+^ core, with vertex‐capping or bridging ligands are not without precedent (for example, [Ag_4_(μ_3_‐I)_4_I_4_]^4−^ and [Ag_4_(TeC_4_H_3_S)_6_]^2−^).^[25]^ Most are based around conventional donor ligands such as halides, S‐ or Te‐based ligands, rather than the unusual low‐valent Ga ligands seen here. However, low oxidation state main‐group ligands based on stanna‐closo‐dodecaborate dianions have been found to stabilize an [Ag_4_]^4+^ cluster in [{Ag(μ_3_‐SnB_11_H_11_)(PMe_3_)}_4_]^4−^.^[26]^ The Ag−Ag bonds within **4** (av. 2.87 Å) are amongst the shortest found for structures of this type on the Cambridge Crystallographic Database (av. for ten structures containing [Ag_4_]^4+^ tetrahedra is 3.110 Å). Compound **4** contains only the fourth example of structurally authenticated Ag−Ga bonds. The average Ag–[Ga(OTf)_3_]^2−^ bond length of 2.47 Å is shorter than the Ag−Ga bond lengths found in [Ag(GaCp*)_4_]^+^ (average 2.519 Å),^[27]^ but longer than those found within the complexes [Ag{Ga(^Dip^DAB)}(IMes)] (2.416 Å) and [Ag{Ga(^Dip^DAB)}(IDip)] (2.411 Å); where ^Dip^DAB=[(DipNCH)_2_]^2−^, IMes=:C(MesNCH)_2_, IDip=:C(DipNCH)_2_, Dip=2,6‐diisopropylphenyl and Mes=mesityl.^[28]^ The Ga‐containing N‐heterocyclic carbene analogue [Ga(^Dip^DAB)]^−^ is known to be a strong donor ligand, GaCp* less so, and so based on Ag‐Ga bond lengths alone it appears that the donor properties of [Ga(OTf)_3_]^2−^ lie in between that of GaCp* and [Ga(^Dip^DAB)]^−^.

Only one example of a complex that could be considered to involve the [Ga(OTf)_3_]^2−^ motif has previously been reported, [(Cp*Ga)Cu(μ‐GaCp*)_3_Cu{Ga(OTf)_3_}] (**5**; Figure 8).^[29]^ The authors formulate this as a Lewis acid/base adduct between [(Cp*Ga)Cu(μ‐GaCp*)_3_Cu], comprising two Cu^0^ centers, and Ga^III^ triflate. An alternative view, in light of our analysis of compound **4**, is that **5** could involve two Cu^I^ centers, with the [(Cp*Ga)Cu(μ‐GaCp*)_3_Cu]^2+^ ion coordinated by [Ga(OTf)_3_]^2−^. As noted above, for Ga_3_X_5_L_3_, it is challenging to unambiguously assign oxidation states to such species.^[14b]^ However, some features of **5** suggest that a Ga^I^/Cu^I^ model is a plausible alternative formulation. The Cu‐{Ga(OTf)_3_} distance (2.2906 Å) is shorter than the terminal Cu‐GaCp* distance (2.3268 Å). This is consistent with the shorter Ag−Ga distances in Ag–[Ga(OTf)_3_]^2−^ complexes compared to Ag–GaCp* complexes, as described above. The average Ga−OTf distances for the “Ga(OTf)_3_” unit in **5** (1.975 Å) are more similar to those in **4** (1.996 Å) than to the shorter Ga‐OTf distances in Ga^III^ systems such as [Ga(OTf)_3_(THF)_3_] (1.945 Å).^[13]^ Finally, NBO calculations carried out on **5** and the dianionic cluster in **4** are consistent with a similar Ga^I^ donor model for the “Ga(OTf)_3_” units in both. In particular, the gallium center contributes 72 % of the electron density to the Ga−Cu bond in **5** and 85 % to the Ga−Ag bond in **4**.

**Figure 8:**
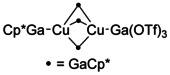
The structure of [(Cp*Ga)Cu(μ‐GaCp*)_3_Cu{Ga(OTf)_3_}] (**5**).^[29]^

In conclusion, a number of low‐valent gallium species have been isolated from “GaOTf” solutions in aromatic solvents. These suggest that “GaOTf” in toluene is best considered a mixed‐valence compound, even in the presence of more π‐basic arenes, such as hexamethybenzene. However, the reaction of “GaOTf” with [18]‐crown‐6 leads to the isolation of a relatively stable univalent gallium crown ether complex. This simplification in speciation, compared to the crown‐free system, may be linked to improved catalytic behavior seen by Schneider et al. Additionally, the bimetallic silver(I)/gallium(I) cluster anion [Ag_4_{Ga(OTf)_3_}_4_(μ‐Ga)_6_(OTf)_4_]^2−^ has been identified at short reaction times. Thus, while “GaOTf” solutions are very promising reagents for the emerging field of low‐valent Ga catalysis, care is required when considering catalytic mechanisms, as the complex speciation in these systems mean that it may not always be obvious what the catalytically active metal and/or oxidation state will be.

## Conflict of interest

The authors declare no conflict of interest.

## Supporting information

As a service to our authors and readers, this journal provides supporting information supplied by the authors. Such materials are peer reviewed and may be re‐organized for online delivery, but are not copy‐edited or typeset. Technical support issues arising from supporting information (other than missing files) should be addressed to the authors.

Supplementary
