# Supplementary material for: Insights into the Composition and Structural Chemistry of Gallium(I) Triflate
Source: Angew Chem Int Ed Engl. 2020 Nov 23;60(3):1567–72. doi: 10.1002/anie.202010837 (PMC7839670; doi:10.1002/anie.202010837)
Supplement: Supplementary file 1 — Supplementary [file ANIE-60-1567-s001.pdf]

Angewandte Chemie
Eine Zeitschrift der Gesellschaft Deutscher Chemiker

Supporting Information

Insights into the Composition and Structural Chemistry of Gallium(I) Triflate

*Josef T. Boronski, Matthew P. Stevens, Bono van IJzendoorn, Adrian C. Whitwood, and John M. Slattery**

anie_202010837_sm_miscellaneous_information.pdf

1. Contents

1. Contents 1
2. Experimental details 2
   2.1. General considerations 2
   2.2. Synthesis of [Ga][Ga(C_6H_5Me)$_2$]$_2$[Ga$_3$(OTf)$_8$] (1) 2
   2.3. Synthesis of [Ga(C_6H_5Me)(C_6Me_6)]$_2$[Ga$_2$(OTf)$_6$].C_6H_5Me (2) 2
   2.4. Synthesis of [Ga(18-crown-6)(OTf)] (3) 3
   2.5. Synthesis of [Ga(C_6H_5Me)$_2$]$_2$[Ag$_4${Ga(OTf)$_3$}$_4$(μ-Ga)$_6$(OTf)$_4$].C_6H_5Me (4) 3
3. Single-crystal X-ray structural data 4
   3.1. Crystal data for [Ga][Ga(C_6H_5Me)$_2$]$_2$[Ga$_3$(OTf)$_8$] (1) 4
   3.2. Crystal data for [Ga(C_6H_5Me)(C_6Me_6)]$_2$[Ga$_2$(OTf)$_6$].C_6H_5Me (2) 6
   3.3. Crystal data for [Ga(18-crown-6)(OTf)] (3) 7
   3.4. Crystal data for [Ga(C_6H_5Me)$_2$]$_2$[Ag$_4${Ga(OTf)$_3$}$_4$(μ-Ga)$_6$(OTf)$_4$].C_6H_5Me (4) 8
4. Computational methods 10
   4.1. General considerations 10
   4.2. Computational data for 3 10
   4.3. Computational data for Cp*Ga 12
   4.4. Computational data for the dianion in 4 – [Ag$_4${Ga(OTf)$_3$}$_4$(μ-Ga)$_6$(OTf)$_4$]$^{2-}$ 13
   4.5. Computational data for [(Cp*Ga)Cu(μ-GaCp*)$_3$Cu{Ga(OTf)$_3$}] (5) 17
5. References 21

2. Experimental details

2.1. General considerations

All manipulations were performed using grease-free Schlenk line or glovebox techniques and a dinitrogen atmosphere. Toluene was dried over sodium, distilled and degassed prior to use. The water content of the solvents used was confirmed to be lower than 10 ppm via Karl Fischer analysis. Gallium metal (Alfa Aesar, pellets 6 mm diameter, 99.99999% (metals basis)), silver(I) triflate (Acros, 99%), [18]-crown-6 (Fluka, 99%) and hexamethylbenzene (Sigma Aldrich, 99%) were used as provided. NMR spectra were acquired on a Bruker AVANCE III 500 spectrometer (Operating Frequencies; ^{1}H 500.23 MHz, ^{19}F 470.68 MHz, ^{71}Ga 152.52 MHz). NMR experiments were performed in 5 mm NMR tubes fitted with PTFE J. Young's taps, by taking *ca.* 0.5 cm^3 aliquots from the reaction mixtures (which used non-deuterated toluene as solvent). Experiments using ultrasonic activation were performed using an Ultrawave SFE590/1 (0.08 KVA) ultrasonic bath.

2.2. Synthesis of $[Ga][Ga(C_6H_5Me)_2]_2[Ga_3(OTf)_8]$ (1)

Within a Young's ampoule, a solution of AgOTf (100 mg, 0.359 mmol) in toluene (*ca.* 15 mL) and a bead of gallium (40 mg, 0.580 mmol, 1.6 equiv.) were allowed to react under ultrasonic activation at *ca.* 45 °C for 1.5 hours. The solution rapidly changed from yellow to brown in colour and a dense black precipitate formed. The precipitate was allowed to settle, and the solution was filtered through a high-porosity glass frit, yielding a pale brown solution. Very pale brown crystals of sufficient quality for X-ray diffraction were obtained by cooling overnight to -20 °C. Manipulation of **1** is hampered by its very high air- and moisture-sensitivity and by its decomposition over time, more rapidly in solution than in the solid state, and on removal of the solvent, with the formation of black/brown solids that suggest disproportionation and formation of Ga metal. As such, a crystalline yield of **1** is not reported.

A molar ratio of *ca.* 1:2 Ag(I) to Ga(0) was typically used for the preparation of "GaOTf" solutions in our experiments.

^{19}F NMR (470 MHz, C_6H_5Me, 298K): δ = -78.7 (s, $[O_3SCF_3]^-$) ppm.
^{71}Ga NMR (152 MHz, C_6H_5Me, locked and shimmed to a sample of $GaCl_3$ in D_2O, 298 K): δ = -692 ppm (s, $[Ga(C_6H_5Me)_2]^+$). No additional Ga signals were observed, i.e. for the Ga environments in the anion of **1**, presumably due to fast quadrupolar relaxation in these low-symmetry environments, and the signal broadening associated with this.

2.3. Synthesis of $[Ga(C_6H_5Me)(C_6Me_6)]_2[Ga_2(OTf)_6].C_6H_5Me$ (2)

Within a Young's ampoule, a solution of AgOTf (100 mg, 0.359 mmol) and hexamethylbenzene (250 mg, 1.54 mmol, 4.3 equiv.) in dry toluene (*ca.* 15 mL) and a bead of gallium (54 mg, 0.783 mmol, 2.2 equiv.) were allowed to react under ultrasonic activation at *ca.* 45 °C for 1.5 hours. The solution rapidly changed from yellow to brown in colour and a dense black precipitate formed. The precipitate was allowed to settle, and the solution was filtered, yielding a colourless solution. Slightly brown crystals of sufficient quality for X-ray diffraction were obtained by cooling overnight to -20 °C. Unreacted hexamethylbenzene also crystallises from solution under these conditions, thus, a crystalline yield for **2** is not reported. Manipulation and further purification of **2** is hampered by its high air- and moisture-sensitivity and by its decomposition over time, more rapidly in solution than in the solid state, and on removal of the solvent, with the formation of black/brown solids that suggest disproportionation and formation of Ga metal.

^{19}F NMR (470 MHz, C_6H_5Me, 298K): δ = -77.6 (s, $[O_3SCF_3]^-$) ppm.
^{71}Ga NMR (152 MHz, C_6H_5Me, locked and shimmed to a sample of $GaCl_3$ in D_2O, 298 K): δ = -706 ppm (s, $[Ga(arene)_2]^+$). No additional Ga signals were observed, i.e. for the Ga environment in the anion of **2**, presumably due to fast quadrupolar relaxation in these low-symmetry environments, and the signal broadening associated with this.

2.4. Synthesis of [Ga(18-crown-6)(OTf)] (3)

Within a Young's ampoule, a solution of AgOTf (100 mg, 0.359 mmol) in dry toluene (*ca.* 15 mL) and a bead of gallium (59 mg, 0.856 mmol, 2.4 equiv.) were allowed to react under ultrasonic activation at *ca.* 45 °C for 1.5 hours. The solution rapidly changed from yellow to brown in colour and a dense black precipitate formed. The precipitate was allowed to settle, and the solution was filtered directly onto a solution of [18]-crown-6 (80 mg, 0.303 mmol, 0.8 equiv.) in *ca.* 5 mL of toluene. In reactions of this type, this often led to the spontaneous formation of colourless, block-like crystals. Storage of the solution overnight at -20 ^{o}C led to the formation of more crystalline material (77 mg, 53% yield based on 18-crown-6).

0.8 equiv. of 18-crown-6 were used in this reaction to account for incomplete formation of Ga(I) species and/or partial decomposition of these during the initial reaction between AgOTf and Ga metal, resulting in sub-stoichiometric amounts of Ga(I) present in solution. The exact conversion of AgOTf to Ga(I) species during these reactions is unknown and likely varies depending on the conditions used. Successful formation of **3** was found to be highly dependent on the concentration of the "GaOTf" solution in toluene. If this concentration was too high, the deposition of a black/brown precipitate (thought to be gallium metal) was observed upon addition of the "GaOTf" solution to 18-crown-6.

^{1}H NMR (500 MHz, C_6H_5Me, 298K): δ = 3.47 (s, 18-crown-6) ppm.
This can be compared to pure 18-crown-6 in neat toluene, which displayed a resonance at 3.36 ppm. The related [Ag(18-crown-6)][OTf] complex displayed a resonance at 3.25 ppm in the same solvent system in our hands.
^{19}F NMR (470 MHz, C_6H_5Me, 298K): δ = -80.5 (s, $[O_3SCF_3]^-$) ppm.
^{71}Ga NMR (152 MHz, C_6H_5Me, locked and shimmed to a sample of $GaCl_3$ in D_2O, 298 K): No signals that could be assigned to **3** were observed in ^{71}Ga NMR spectra of solutions formed when adding "GaOTf" to 18-crown-6. This may be due to the relatively low solubility of **3** in toluene and/or signal broadening, due to its low symmetry. In one experiment, a broad signal at -66 ppm was observed, but this is at a very different chemical shift to that reported by Schneider and Krossing for $[Ga(18\text{-crown-}6)]^+$ (-566 ppm in dioxane, -643 ppm in C_6H_5F) and it is assumed that this relates to a degree of degradation and formation of an unidentified Ga(III) species.
ATR IR (298K, cm^{-1}) ν = 2870 (br, m), 1468 (w), 1450 (w), 1352 (m), 1250 (br, m), 1199 (s), 1100 (br, vs), 1029 (s), 948 (m), 927 (m), 829 (w, br), 733 (w), 633 (s), 570 (w), 515 (m), 466 (w).

2.5. Synthesis of $[Ga(C_6H_5Me)_2]_2[Ag_4\{Ga(OTf)_3\}_4(\mu\text{-}Ga)_6(OTf)_4].C_6H_5Me$ (4)

Crystals of compound **4** were initially observed after a reaction where a "GaOTf" solution in toluene was prepared as described above for the synthesis of **1** (using 116 mg Ga and 215 mg, 0.5 equiv. AgOTf), but where the sample was only sonicated for around 40 minutes. An alternative synthetic approach is given below. In both cases, orange crystals of **4** were formed alongside other species and so characterisation data is not presented. We provide the synthetic details here to give context for the formation of the crystalline material analysed by single-crystal X-ray diffraction (details below).

Within a Young's ampoule, a solution of AgOTf (300 mg, 1.17 mmol, 1 equiv.) in dry toluene (*ca.* 15 mL) and a bead of gallium (158 mg, 2.29 mmol, 2 equiv.) were allowed to react under ultrasonic activation at *ca.* 45 °C for 1.5 hours. The solution rapidly changed from yellow to brown in colour and a dense black precipitate formed. The precipitate was allowed to settle and the solution was filtered directly onto AgOTf (50 mg, 0.195 mmol), leading to the formation of a yellow solution. Storage overnight at -20°C led to the formation of some orange crystals that were individually removed for X-ray diffractometry investigations.

3. Single-crystal X-ray structural data

All single-crystal X-ray crystallography was performed at 110 K. Data sets were obtained on an Agilent SuperNova with $Cu_{K\alpha}$ radiation (λ = 1.54184 Å). Crystal preparation was carried out at -23 °C, in mineral oil and within a Petri dish under a blanket of argon gas. Using Olex2, the structure was solved with the Superflip structure solution program using Charge Flipping and refined with the ShelXL refinement package using Least Squares minimisation. All non-hydrogen atoms were refined anisotropically. Unless stated otherwise, hydrogen atoms were placed using a "riding model" and included in the refinement at calculated positions.

3.1. Crystal data for $[Ga][Ga(C_6H_5Me)_2]_2[Ga_3(OTf)_8]$ (1)

CCDC deposition number 1994418

Identification code	jms1701
Empirical formula	$C_{39.5}H_{36}F_{24}Ga_6O_{24}S_8$
Formula weight	2025.48
Temperature/K	110.05(10)
Crystal system	monoclinic
Space group	$P2_1/c$
a/Å	10.30441(15)
b/Å	31.4481(4)
c/Å	21.6431(4)
α/°	90
β/°	99.0979(14)
γ/°	90
Volume/Å^3	6925.30(18)
Z	4
ρ_{calc}g/cm^3	1.943
μ/mm^{-1}	6.106
F(000)	3980.0
Crystal size/mm^3	0.115 × 0.062 × 0.046
Radiation	CuKα (λ = 1.54184)
2Θ range for data collection/°	6.98 to 134.156
Index ranges	$-12 \le h \le 12$, $-31 \le k \le 37$, $-18 \le l \le 25$
Reflections collected	25993
Independent reflections	12366 [R_{int} = 0.0578, R_{sigma} = 0.0783]
Data/restraints/parameters	12366/48/966
Goodness-of-fit on F^2	1.059
Final R indexes [I>=2σ (I)]	R_1 = 0.0784, wR_2 = 0.2100
Final R indexes [all data]	R_1 = 0.1020, wR_2 = 0.2303
Largest diff. peak/hole / e Å^{-3}	2.81/-1.40

As noted in the main section, compound **1** contains a Ga^+ ion in an unusual coordination environment, which we describe as a "naked" cation, as it is not coordinated by arenes, as is commonly seen. This ion is, however, stabilized by interactions with two neighbouring $[Ga_3(OTf)_8]^{3-}$ ions within the crystal lattice. This coordination environment, and details of the Ga···O and Ga···F contacts are shown in Fig. S1.

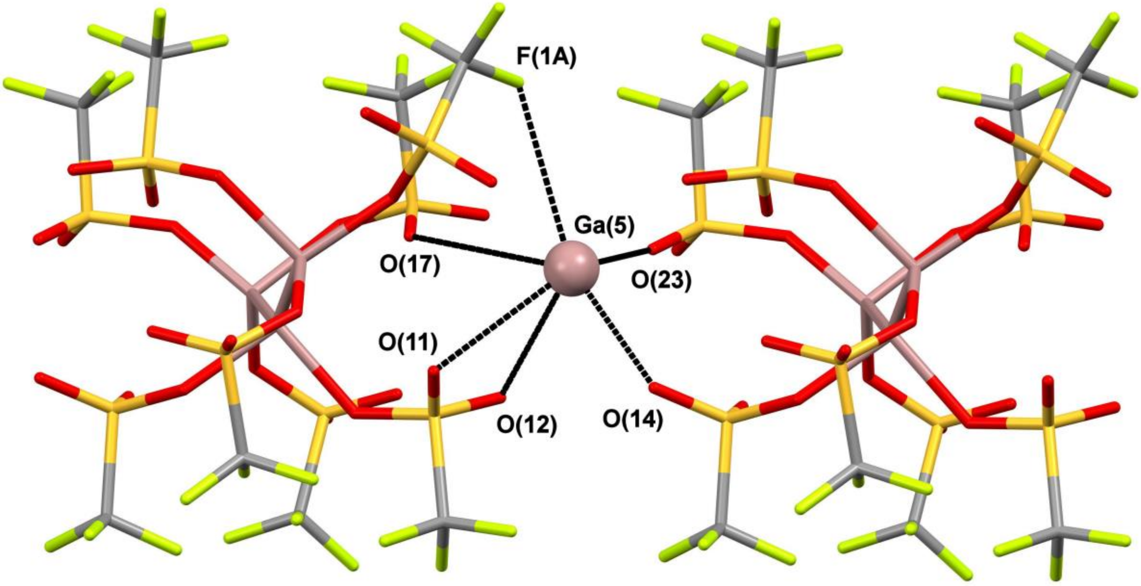

Figure S1. Anion contacts less than the sum of the VdW radii (in Å) to the "naked" gallium(I) cation in **1**. Ga(5)-F(1A) 3.281, Ga(5)-O(11) 2.962, Ga(5)-O(12) 2.792, Ga(5)-O(14) 2.456, Ga(5)-O(17) 3.333, Ga(5)-O(23) 2.449.

3.2. Crystal data for $[Ga(C_6H_5Me)(C_6Me_6)]_2[Ga_2(OTf)_6].C_6H_5Me$ (2)

CCDC deposition number 1994419

Identification code	jms1702_twin1_hklf4
Empirical formula	$C_{51}H_{60}F_{18}Ga_4O_{18}S_6$
Formula weight	1774.23
Temperature/K	109.95(10)
Crystal system	monoclinic
Space group	$P2_1/n$
a/Å	12.0600(16)
b/Å	15.826(2)
c/Å	18.253(4)
α/°	90
β/°	91.894(17)
γ/°	90
Volume/Å^3	3481.9(10)
Z	2
ρ_{calc}g/cm^3	1.692
μ/mm^{-1}	4.460
F(000)	1784.0
Crystal size/mm^3	0.227 × 0.106 × 0.069
Radiation	CuKα (λ = 1.54184)
2Θ range for data collection/°	7.394 to 84.332
Index ranges	$-7 \le h \le 10$, $-13 \le k \le 13$, $-15 \le l \le 15$
Reflections collected	3185
Independent reflections	3185 [R_{int} = ?, R_{sigma} = 0.0693]
Data/restraints/parameters	3185/165/418
Goodness-of-fit on F^2	1.042
Final R indexes [I>=2σ (I)]	R_1 = 0.0861, wR_2 = 0.2279
Final R indexes [all data]	R_1 = 0.1144, wR_2 = 0.2444
Largest diff. peak/hole / e Å^{-3}	0.57/-0.45

Refinement special details

The crystal diffracted poorly which lead to a low-quality structure using data to 1.15 angstroms resolution. The crystal was also non-merohedrally twinned which was modelled using two components in a refined ratio of 0.567:0.463(4). The following restraints and constraints were applied to the model:

The asymmetric unit contained half of the bis-gallium hexatriflate anion, the two halves being related by a centre of inversion. For one triflate (S3, O7-9, C3, F7-9), the C-S bond was restrained to be 1.83 angstroms and the ADP of the carbon and fluorines restrained to be approximatelyisotropic.

For the hexamethylbenzene (C4-C15), the aromatic ring was constrained to be a regular hexagon with C-C bond lengths of 1.39 angstroms. The ADP of the carbons were restrained to be approximately isotropic.

For the toluene (C16-C22) complexed to the gallium, the aromatic ring was constrained to be a regular hexagon with C-C bond lengths of 1.39 angstroms. The methyl carbon was initially placed at the site of highest electron density although it is probably that there are othe sites for this atom with the structure. The C-Me bond length (C16-C17) was restrained to be 1.5 angstroms and the methyl carbon was fixed in one position with the

C(methyl)-C(ortho) distances restrained to be equal. The ADP of all carbon atoms were restrained to be approximately isotropic.

The other toluene was disordered about a centre of inversion with half a molecule in the asymmetric unit. The ADP of the carbons were restrained to be approximately isotropic and the ADP of C23 and C24 constrained to be equal.

3.3. Crystal data for [Ga(18-crown-6)(OTf)] (3)

CCDC deposition number 1994420

Identification code	jms1713
Empirical formula	$C_{13}H_{24}F_3GaO_9S$
Formula weight	483.10
Temperature/K	110.05(10)
Crystal system	monoclinic
Space group	$P2_1/n$
a/Å	9.30472(14)
b/Å	11.63093(16)
c/Å	18.6168(3)
α/°	90
β/°	101.8506(14)
γ/°	90
Volume/Å^3	1971.81(5)
Z	4
ρ_{calc}g/cm^3	1.627
μ/mm^{-1}	3.565
F(000)	992.0
Crystal size/mm^3	0.376 × 0.287 × 0.117
Radiation	CuKα (λ = 1.54184)
2Θ range for data collection/°	9.02 to 134.158
Index ranges	$-11 \le h \le 8$, $-13 \le k \le 13$, $-22 \le l \le 22$
Reflections collected	15337
Independent reflections	3521 [R_{int} = 0.0211, R_{sigma} = 0.0172]
Data/restraints/parameters	3521/0/244
Goodness-of-fit on F^2	1.083
Final R indexes [I>=2σ (I)]	R_1 = 0.0214, wR_2 = 0.0548
Final R indexes [all data]	R_1 = 0.0240, wR_2 = 0.0564
Largest diff. peak/hole / e Å^{-3}	0.26/-0.34

3.4. Crystal data for $[Ga(C_6H_5Me)_2]_2[Ag_4\{Ga(OTf)_3\}_4(\mu\text{-}Ga)_6(OTf)_4].C_6H_5Me$ (4)

CCDC deposition number 1994421

Identification code	jms1717
Empirical formula	$C_{51}H_{39}Ag_4F_{48}Ga_{12}O_{48}S_{16}$
Formula weight	4112.90
Temperature/K	110.00(10)
Crystal system	monoclinic
Space group	C2/c
a/Å	20.5252(2)
b/Å	20.5698(3)
c/Å	28.1791(3)
α/°	90
β/°	101.3254(12)
γ/°	90
Volume/Å^3	11665.5(2)
Z	4
ρ_{calc}g/cm^3	2.342
μ/mm^{-1}	12.403
F(000)	7908.0
Crystal size/mm^3	0.584 × 0.124 × 0.038
Radiation	CuKα (λ = 1.54184)
2Θ range for data collection/°	7.316 to 134.15
Index ranges	$-22 \leq h \leq 24$, $-18 \leq k \leq 24$, $-30 \leq l \leq 33$
Reflections collected	21239
Independent reflections	10405 [R_{int} = 0.0371, R_{sigma} = 0.0424]
Data/restraints/parameters	10405/129/879
Goodness-of-fit on F^2	1.036
Final R indexes [I>=2σ (I)]	R_1 = 0.0625, wR_2 = 0.1727
Final R indexes [all data]	R_1 = 0.0692, wR_2 = 0.1828
Largest diff. peak/hole / e Å^{-3}	3.15/-0.89

Refinement special details

The structure exhibited considerable disorder which was modelled as follows:

Five triflate anions were each modelled in two positions with the following refined occupancies, restraints and constraints:

1. Occupancies 0.692:0.308(4), restraints - minor component C-S 1.81 angstroms, C-F 1.33 angstroms, adjacent F-F distances equal, S-F distances equal, constraints - equal ADP for proximal S2A & S2B, F4B & O6A, F5A & F5B, F6A & O6B, F4A & F6B, C2A & C2B.

2. Occupancies 0.856:0.144(7), restraints - S4B-O12B 1.41 angstroms, F10B-C4B, F11B-C4B and F12B-C4B, C4a-F12A 1.33 angstroms, C4B- S4B 1.81 angstroms; for minor component, the adjacent F-F distances equal, S-F distances equal, O10-O12B & O11B-O12B equal, C4B-O10, C4B-O11B C4B-O12B equal. Constraints - equal ADP for C4B O12A & F11B, S4B & S4A, O12B & F12A, F10A & F10B, O11A & O11B, F11A & F12B

3. Occupancies 0.888:0.112(4), restraints - C5B- S5B 1.81 angstroms, C5B-F13B, C5B-F14B, C5B-F15B 1.33 angstroms, for minor component, the adjacent F-F distances equal, S-F distances equal, S5B-O15B and S5B-O14 equal, O13-O15B & O13-O14 equal, C5B-O13, C5B-O14 and C5B-O13B equal. Constraints - equal ADP for S5A, S5B & C5B, F13A & O15B, O15A & F15B, F15A & F14B, F14A & F13B

4. Occupancies 0.718:0.282(4), restraints - C6B- S6B 1.81 angstroms. Constraints - equal ADP for C6A & O18B, C6B & O18A

5. Occupancies 0.641:0.359(9), restraints - F21B-C7B, C7B-F20B & F19B-C7B equal, C-S equal, S7A-O21A & S7B-O21B equal, O19-C7B, C7B-O21B, C7B-O20 equal. Constraints - equal ADP for F21A & O21B, O21B & C7A, O21A C7B & F21B.

The gallium bis(toluene) cation was considerably disordered. The gallium was modelled in two positions each with 50% occupancy. One toluene was modelled in two positions with occupancy of each set at 50%. The other was modelled in 3 positions with occupancies of 50%, 25% and 25%. The toluenes were modelled as follows:

The phenyl rings were constrained to be regular hexagons with a C-C bond length of 1.39 angstroms. The carbons were modelled isotropically with Uiso fixed at 0.2. The C(ipso)-C(Me) bond length was restrained to be 1.48 angstroms. C(Me), C(ipso) and C(ortho)s were restrained to be in the same plane.

There was an additional toluene of crystallisation disordered about an inversion centre equivalent to 50% occupancy in each asymmetric unit. The phenyl ring was constrained to be a regular hexagon with a C-C bond length of 1.39 angstroms. The C(ipso)-C(Me) bond length was restrained to be 1.48 angstroms. The ADP were restrained to be approximately isotropic. C(Me), C(ipso) and C(ortho)s were restrained to be in the same plane.

4. Computational methods

4.1. General considerations

Geometry optimisations were performed at the (RI-)BP86/SV(P) level, followed by frequency calculations at the same level. All minima were confirmed as such by the absence of imaginary frequencies. In calculations involving Ag, a 28 electron quasi-relativistic ECP replaced the core electrons at the metal. No symmetry constraints were applied during optimisations. These steps were performed using the TURBOMOLE 6.4 package using the resolution of identity (RI) approximation.[1]

Single-point calculations on the (RI-)BP86/SV(P) optimised geometries were performed using the hybrid PBE0 functional and the flexible def2-TZVPP basis set using GAUSSIAN 09 revision D.01 in order to prepare input files for use by the NBO software.[2] NBO analysis was performed using NBO 7.0.5.[3]

Structures were visualised and modified using Facio,[4] Jmol,[5] and gOpenMol.

4.2. Computational data for 3

(RI-)BP86/SV(P) SCF Energy (a.u.) = -3808.245386

(RI-)PBE0/def2-TZVPP//(RI-)BP86/SV(P) SCF Energy (a.u.) = -3808.035187

XYZ coordinates

51

C	4.24901	2.30065	9.89292
F	5.39279	2.67192	9.29630
F	3.30256	2.14697	8.94537
F	4.44394	1.11064	10.49286
Ga	0.79083	3.26374	12.91065
O	2.44113	2.93511	11.68383
O	4.82792	3.63353	12.14911
O	3.47231	4.83030	10.35214
S	3.72236	3.60683	11.15785
C	-1.16469	2.05634	9.99465
H	-1.47201	2.05138	8.91712
H	-2.09610	2.05163	10.61636
C	-0.34940	0.80457	10.26791
H	-0.90304	-0.08484	9.86835
H	0.62522	0.88238	9.72629
C	0.70467	-0.42220	11.98915
H	1.66453	-0.36461	11.41911
H	0.20398	-1.39279	11.73477
C	1.01757	-0.39964	13.47550
H	0.06920	-0.35100	14.06903
H	1.54048	-1.35414	13.74166
C	2.41161	0.73856	15.04242
H	2.97420	-0.20957	15.24287
H	1.61752	0.83833	15.82572
C	3.37834	1.90616	15.13239
H	3.96243	1.81706	16.08484
H	4.09237	1.86942	14.27483
C	3.49482	4.26883	15.15300
H	4.24658	4.22124	14.32970
H	4.02929	4.32356	16.13659

C	2.64994	5.51658	14.98227
H	1.81283	5.52835	15.72558
H	3.29300	6.41306	15.17075
C	1.46805	6.74404	13.30954
H	2.10427	7.63255	13.55114
H	0.51087	6.83748	13.88266
C	1.19842	6.73390	11.81697
H	0.80116	7.73660	11.51194
H	2.15273	6.53712	11.27439
C	0.05700	5.53657	10.12460
H	1.02516	5.27609	9.63418
H	-0.33795	6.47843	9.66309
C	-0.95098	4.42515	9.89589
H	-1.88334	4.62589	10.48287
H	-1.22588	4.40830	8.80983
O	-0.36837	3.19113	10.27245
O	-0.13530	0.66980	11.66163
O	1.85051	0.71037	13.74394
O	2.64955	3.12455	15.11675
O	2.14981	5.54859	13.65479
O	0.24733	5.71830	11.52124

Vibrational frequencies (first 50 modes)

# mode	symmetry	wave number	IR intensity	selection rules	
#		cm**(-1)	km/mol	IR	RAMAN
1		0.00	0.00000	-	-
2		0.00	0.00000	-	-
3		0.00	0.00000	-	-
4		0.00	0.00000	-	-
5		0.00	0.00000	-	-
6		0.00	0.00000	-	-
7	a	20.42	0.33177	YES	YES
8	a	25.04	0.07982	YES	YES
9	a	31.27	0.27985	YES	YES
10	a	37.54	1.04460	YES	YES
11	a	44.39	0.33517	YES	YES
12	a	51.10	1.05388	YES	YES
13	a	54.49	0.80345	YES	YES
14	a	64.04	2.59304	YES	YES
15	a	68.45	4.98762	YES	YES
16	a	75.51	14.86811	YES	YES
17	a	79.63	8.14586	YES	YES
18	a	81.61	10.76608	YES	YES
19	a	86.66	7.66708	YES	YES
20	a	88.38	1.10795	YES	YES
21	a	109.89	2.82316	YES	YES
22	a	124.44	4.05436	YES	YES
23	a	129.59	2.72397	YES	YES
24	a	152.80	3.45916	YES	YES
25	a	158.69	27.66490	YES	YES
26	a	162.82	1.82883	YES	YES
27	a	172.53	7.84443	YES	YES
28	a	175.39	9.98908	YES	YES
29	a	190.07	0.08762	YES	YES
30	a	204.84	2.96188	YES	YES
31	a	227.51	6.51415	YES	YES
32	a	231.69	8.18608	YES	YES
33	a	233.63	12.52589	YES	YES
34	a	237.90	1.93529	YES	YES
35	a	250.78	6.34527	YES	YES

36	a	256.85	11.12774	YES	YES
37	a	278.04	1.83397	YES	YES
38	a	290.27	9.50577	YES	YES
39	a	311.04	0.01039	YES	YES
40	a	313.23	0.68231	YES	YES
41	a	316.93	0.55013	YES	YES
42	a	341.20	23.84143	YES	YES
43	a	353.46	0.01053	YES	YES
44	a	356.98	0.08200	YES	YES
45	a	358.88	0.19501	YES	YES
46	a	451.85	14.42265	YES	YES
47	a	481.34	8.24731	YES	YES
48	a	515.03	0.91058	YES	YES
49	a	515.36	0.61089	YES	YES
50	a	521.08	0.49678	YES	YES

4.3. Computational data for Cp*Ga

(RI-)BP86/SV(P) SCF Energy (a.u.) = -2314.713566

(RI-)PBE0/def2-TZVPP//(RI-)BP86/SV(P) SCF Energy (a.u.) = -2314.389390

XYZ coordinates

26

C	-2.10788	4.25554	1.03954
C	-2.56884	3.54770	-0.12691
C	-3.34293	2.41721	0.31573
C	-2.59635	3.56166	2.20317
C	-3.36023	2.42603	1.75557
Ga	-4.53666	4.42427	1.01111
C	-2.21943	3.87944	-1.55432
H	-2.06644	4.97041	-1.70321
H	-1.27517	3.37400	-1.86842
H	-3.01072	3.55581	-2.26460
C	-1.19638	5.45530	1.04080
H	-0.12291	5.15001	1.02770
H	-1.35802	6.10351	0.15227
H	-1.34116	6.08829	1.94285
C	-2.28544	3.90974	3.63570
H	-1.36021	3.39398	3.98720
H	-2.11936	5.00017	3.77268
H	-3.10374	3.61074	4.32607
C	-3.98601	1.37642	2.63668
H	-3.28350	0.52857	2.81911
H	-4.26823	1.78197	3.63218
H	-4.90387	0.94287	2.18356
C	-3.94659	1.35557	-0.56633
H	-3.23069	0.51677	-0.73847
H	-4.86166	0.91133	-0.11809
H	-4.22627	1.75229	-1.56620

Vibrational frequencies (first 50 modes)

$vibrational spectrum

# mode	symmetry	wave number	IR intensity	selection rules	
#		cm**(-1)	km/mol	IR	RAMAN
1		0.00	0.00000	-	-
2		0.00	0.00000	-	-
3		0.00	0.00000	-	-
4		0.00	0.00000	-	-
5		0.00	0.00000	-	-

6		0.00	0.00000	-	-
7	a	101.07	0.00009	YES	YES
8	a	102.20	0.00121	YES	YES
9	a	111.38	0.00606	YES	YES
10	a	111.56	0.00535	YES	YES
11	a	113.73	0.00007	YES	YES
12	a	122.69	0.34017	YES	YES
13	a	123.42	0.34486	YES	YES
14	a	145.66	0.00001	YES	YES
15	a	146.08	0.00006	YES	YES
16	a	148.92	0.00049	YES	YES
17	a	277.09	1.00572	YES	YES
18	a	277.81	1.06565	YES	YES
19	a	281.16	0.04444	YES	YES
20	a	283.21	0.03936	YES	YES
21	a	322.71	77.52782	YES	YES
22	a	368.93	2.23293	YES	YES
23	a	370.31	2.27844	YES	YES
24	a	536.52	0.00006	YES	YES
25	a	536.58	0.00008	YES	YES
26	a	543.29	0.00198	YES	YES
27	a	587.44	1.87396	YES	YES
28	a	591.35	0.00162	YES	YES
29	a	591.40	0.00040	YES	YES
30	a	798.04	3.40907	YES	YES
31	a	798.87	3.68762	YES	YES
32	a	932.88	0.01958	YES	YES
33	a	934.35	0.01521	YES	YES
34	a	1009.93	8.37568	YES	YES
35	a	1010.50	8.38041	YES	YES
36	a	1017.27	0.54869	YES	YES
37	a	1019.56	0.00531	YES	YES
38	a	1019.89	0.00168	YES	YES
39	a	1053.79	2.75698	YES	YES
40	a	1054.06	3.16742	YES	YES
41	a	1084.90	0.01436	YES	YES
42	a	1160.13	0.00207	YES	YES
43	a	1160.75	0.00335	YES	YES
44	a	1355.51	0.00869	YES	YES
45	a	1356.02	0.00013	YES	YES
46	a	1365.04	0.23551	YES	YES
47	a	1365.48	0.19637	YES	YES
48	a	1369.77	11.04051	YES	YES
49	a	1395.95	0.00132	YES	YES
50	a	1396.45	0.00350	YES	YES

4.4. Computational data for the dianion in 4 – $[Ag_4\{Ga(OTf)_3\}_4(\mu\text{-}Ga)_6(OTf)_4]^{2-}$

(RI-)BP86/SV(P) SCF Energy (a.u.) = -35214.179092

(RI-)PBE0/def2-TZVPP//(RI-)BP86/SV(P) SCF Energy (a.u.) = -35212.320628

XYZ coordinates

142

Ag	-1.42217	-0.79588	0.41072
Ag	-0.41817	1.26538	-1.42604
Ga	1.93725	0.51675	-2.60459
Ga	-3.48509	-2.21995	0.59356
Ga	-0.65553	2.76994	-3.43618

Ga	-2.56535	-0.14214	-1.97268
Ga	0.00018	3.51360	0.00004
Ga	0.00015	-3.05732	0.00009
S	-4.57885	-1.52017	3.54394
S	1.40913	1.65462	-5.64999
S	-2.23217	-3.25370	-3.01703
S	-0.63195	5.76680	-2.37822
S	-5.70610	-0.92381	-1.30362
S	-3.43799	1.79067	-4.74510
S	3.03042	3.36531	-1.65514
S	-2.51452	-5.18830	0.92949
O	0.47220	2.76174	-5.16346
O	-3.82134	-0.23125	3.28857
O	1.48090	0.50524	-4.64125
O	-4.58660	0.09426	-1.51509
O	-4.49827	-2.48069	2.34840
O	-3.03345	-1.95460	-2.76527
O	-5.25272	-2.08451	-0.40751
F	-2.31813	-2.65825	4.37979
O	-0.52147	4.79481	-3.56200
O	-3.14200	0.54555	-3.93269
O	-0.95001	5.03270	-1.08134
O	-2.37430	2.88147	-4.53850
F	-4.14040	-3.62013	5.11073
O	2.86454	2.33182	-2.78977
O	-3.51937	-4.22592	0.27138
F	-0.79186	0.49036	-6.62959
O	-3.10883	-4.41828	-3.19858
O	-1.09240	-4.66235	0.83992
O	2.66814	2.12099	-6.23694
F	1.07744	-0.21672	-7.51127
F	4.30617	5.68281	-1.70981
O	-5.92445	-1.36118	4.10369
F	-3.49656	-1.71994	5.96555
F	0.29081	1.74739	-8.04991
O	1.74576	4.10803	-1.37948
O	-1.04448	-3.38297	-2.10652
F	5.26281	4.11257	-2.88641
O	-6.49224	-1.32452	-2.47242
F	-1.87770	-6.40736	-1.38048
O	-2.90840	-5.71655	2.24124
F	-0.67020	-1.82309	-4.63956
O	0.38363	6.82269	-2.35304
F	-3.28580	5.74045	-2.73139
F	-5.28813	3.68582	-4.62387
O	3.79496	2.88671	-0.48946
F	-2.40138	-2.72332	-5.62485
C	-3.55664	-2.44752	4.84461
F	-6.25236	0.35634	0.97672
O	-3.80277	1.55787	-6.14674
F	3.46766	5.10128	-3.64098
F	-2.51278	7.53719	-1.76041
F	-0.69881	-3.96694	-5.06426
F	-7.28194	1.15336	-0.77871
C	0.41856	0.85687	-7.05743
C	-6.87477	0.03870	-0.16138
F	-2.22228	7.24544	-3.90438
F	-7.93909	-0.72843	0.11081
F	-5.97429	1.72845	-3.94684
C	4.08818	4.65935	-2.54664
C	-1.44814	-2.91523	-4.70659

C	-4.95204	2.58922	-3.92671
C	-2.28659	6.62742	-2.71582
F	-3.81796	-6.97726	-0.55250
C	-2.54892	-6.66942	-0.26458
F	-1.97671	-7.70927	0.36459
F	-4.67149	2.93270	-2.66837
Ag	1.42245	-0.79583	-0.41085
Ag	0.41846	1.26539	1.42593
Ga	-1.93682	0.51669	2.60448
Ga	3.48541	-2.21986	-0.59348
Ga	0.65583	2.76989	3.43617
Ga	2.56566	-0.14205	1.97264
S	4.57899	-1.52047	-3.54403
S	-1.40865	1.65416	5.64993
S	2.23248	-3.25376	3.01691
S	0.63191	5.76679	2.37837
S	5.70647	-0.92370	1.30358
S	3.43833	1.79080	4.74507
S	-3.03007	3.36522	1.65530
S	2.51481	-5.18819	-0.92936
O	-0.47184	2.76143	5.16352
O	3.82178	-0.23137	-3.28868
O	-1.48023	0.50487	4.64106
O	4.58695	0.09434	1.51508
O	4.49848	-2.48082	-2.34834
O	3.03367	-1.95459	2.76523
O	5.25310	-2.08442	0.40750
F	2.31723	-2.65630	-4.38012
O	0.52148	4.79473	3.56211
O	3.14240	0.54565	3.93268
O	0.95023	5.03279	1.08150
O	2.37461	2.88157	4.53844
F	4.13861	-3.62159	-5.10874
O	-2.86403	2.33176	2.78995
O	3.51959	-4.22583	-0.27114
F	0.79189	0.48873	6.62913
O	3.10918	-4.41831	3.19851
O	1.09263	-4.66238	-0.83968
O	-2.66773	2.12034	6.23690
F	-1.07754	-0.21620	7.51227
F	-4.30597	5.68263	1.71003
O	5.92452	-1.36184	-4.10405
F	3.49809	-1.72143	-5.96613
F	-0.28897	1.74761	8.04917
O	-1.74546	4.10799	1.37953
O	1.04487	-3.38311	2.10631
F	-5.26243	4.11233	2.88670
O	6.49266	-1.32438	2.47237
F	1.87830	-6.40752	1.38055
O	2.90867	-5.71625	-2.24119
F	0.67190	-1.82208	4.63978
O	-0.38383	6.82252	2.35314
F	3.28578	5.74092	2.73150
F	5.28859	3.68584	4.62402
O	-3.79466	2.88653	0.48968
F	2.40122	-2.72573	5.62522
C	3.55637	-2.44786	-4.84434
F	6.25266	0.35643	-0.97679
O	3.80310	1.55805	6.14671
F	-3.46730	5.10116	3.64115
F	2.51234	7.53776	1.76103

F	0.69702	-3.96628	5.06281
F	7.28223	1.15353	0.77861
C	-0.41800	0.85653	7.05736
C	6.87510	0.03883	0.16131
F	2.22195	7.24536	3.90492
F	7.93944	-0.72825	-0.11088
F	5.97455	1.72855	3.94656
C	-4.08786	4.65919	2.54685
C	1.44814	-2.91544	4.70634
C	4.95237	2.58940	3.92669
C	2.28638	6.62768	2.71620
F	3.81844	-6.97732	0.55224
C	2.54936	-6.66945	0.26453
F	1.97708	-7.70921	-0.36467
F	4.67173	2.93313	2.66843

Vibrational frequencies (first 50 modes)

$vibrational spectrum

# mode	symmetry	wave number	IR intensity	selection rules	
#		cm**(-1)	km/mol	IR	RAMAN
1		0.00	0.00000	-	-
2		0.00	0.00000	-	-
3		0.00	0.00000	-	-
4		0.00	0.00000	-	-
5		0.00	0.00000	-	-
6		0.00	0.00000	-	-
7	a	7.82	4.64756	YES	YES
8	a	11.07	0.02130	YES	YES
9	a	11.51	0.08338	YES	YES
10	a	12.83	0.07388	YES	YES
11	a	12.86	0.26268	YES	YES
12	a	14.86	0.00253	YES	YES
13	a	15.34	0.11806	YES	YES
14	a	16.02	0.15505	YES	YES
15	a	16.82	0.04991	YES	YES
16	a	18.05	0.05379	YES	YES
17	a	19.24	0.17002	YES	YES
18	a	19.52	0.00031	YES	YES
19	a	20.40	0.00053	YES	YES
20	a	20.91	0.20827	YES	YES
21	a	21.01	0.00080	YES	YES
22	a	22.15	0.04723	YES	YES
23	a	23.00	0.00351	YES	YES
24	a	23.40	0.14005	YES	YES
25	a	23.58	0.11600	YES	YES
26	a	24.41	0.12924	YES	YES
27	a	24.43	0.07693	YES	YES
28	a	25.28	0.22033	YES	YES
29	a	27.14	0.08343	YES	YES
30	a	27.21	0.04053	YES	YES
31	a	28.21	0.44440	YES	YES
32	a	28.94	0.03193	YES	YES
33	a	30.32	0.04877	YES	YES
34	a	30.40	0.02515	YES	YES
35	a	31.18	0.01468	YES	YES
36	a	31.40	0.04420	YES	YES
37	a	31.80	0.09586	YES	YES
38	a	32.00	0.00824	YES	YES
39	a	32.37	0.30484	YES	YES
40	a	34.31	0.00101	YES	YES

41	a	34.56	0.20966	YES	YES
42	a	34.94	0.03042	YES	YES
43	a	35.90	0.40566	YES	YES
44	a	37.84	0.31275	YES	YES
45	a	37.95	0.03761	YES	YES
46	a	38.02	0.01995	YES	YES
47	a	39.61	0.08514	YES	YES
48	a	40.02	0.00079	YES	YES
49	a	40.27	0.05620	YES	YES
50	a	41.17	0.22169	YES	YES

4.5. Computational data for [(Cp*Ga)Cu(μ-GaCp*)$_3$Cu{Ga(OTf)$_3$}] (5)

(RI-)BP86/SV(P) SCF Energy (a.u.) = -17348.091435

(RI-)PBE0/def2-TZVPP//(RI-)BP86/SV(P) SCF Energy (a.u.) = -17346.219507

XYZ coordinates

131

Ga	1.22026	3.32328	21.80971
Ga	-2.30154	3.72343	23.77975
Ga	-2.12246	5.00690	20.17361
Ga	-0.31116	6.91729	22.95079
Ga	-4.56429	7.23129	22.91400
Cu	-0.68814	4.61648	22.14548
Cu	-2.60680	5.91214	22.50747
S	3.08312	5.06289	19.94063
S	0.20125	0.64651	20.47193
S	2.37443	2.89852	24.72507
F	3.16988	3.01537	18.21748
F	4.21997	4.81406	17.56124
F	5.10206	3.53237	19.09496
F	2.23105	-1.08829	20.46193
F	1.38743	-0.72371	22.44329
F	0.29596	-1.94771	21.00145
F	4.70095	3.92567	23.88518
F	4.62840	3.43154	26.01098
F	4.81798	1.84078	24.52523
O	2.94201	4.03150	21.09434
O	1.76790	5.45166	19.35944
O	4.04703	6.13550	20.27006
O	1.30346	1.73332	20.60953
O	-0.94949	0.88562	21.38725
O	-0.08592	0.32086	19.05820
O	2.14941	2.35384	23.28769
O	1.85722	4.28491	24.89791
O	2.04099	1.89476	25.75934
C	3.95508	4.02443	18.61563
C	1.10044	-0.88112	21.14484
C	4.26492	3.03074	24.78102
C	-2.13430	1.73378	24.91456
C	-3.39252	1.72885	24.19533
C	-4.22081	2.77660	24.74177
C	-3.47601	3.43800	25.78807
C	-2.18688	2.79740	25.89731
C	-1.04181	0.71546	24.75500
H	-1.30187	-0.20993	25.32138
H	-0.06579	1.07569	25.13917

H	-0.90237	0.43433	23.69139
C	-3.78391	0.72007	23.14898
H	-4.11799	-0.23022	23.62800
H	-2.93362	0.47824	22.47695
H	-4.62428	1.08102	22.51811
C	-5.64124	3.07403	24.34654
H	-6.36306	2.55006	25.01654
H	-5.85945	2.74257	23.30921
H	-5.86876	4.16041	24.40537
C	-3.98426	4.53894	26.67821
H	-4.47437	4.12277	27.59007
H	-4.73748	5.17383	26.16488
H	-3.16332	5.20182	27.02609
C	-1.13267	3.09790	26.92848
H	-1.30980	2.49649	27.85098
H	-1.13901	4.16780	27.22804
H	-0.11163	2.85487	26.56918
C	-1.64452	4.21683	18.06471
C	-1.82377	5.65113	17.97413
C	-3.19308	5.95129	18.31565
C	-3.85904	4.70896	18.63434
C	-2.90452	3.63544	18.48057
C	-0.40277	3.47577	17.65938
H	-0.36802	3.37574	16.54868
H	-0.35866	2.45233	18.08373
H	0.51290	4.01427	17.97849
C	-0.78359	6.62405	17.48723
H	-0.73234	6.61609	16.37298
H	0.22652	6.36994	17.87235
H	-1.01469	7.66610	17.79558
C	-3.83347	7.31008	18.25274
H	-4.20797	7.52700	17.22449
H	-3.11976	8.12043	18.51520
H	-4.70173	7.39040	18.93920
C	-5.31592	4.54388	18.97364
H	-5.92966	4.40426	18.05260
H	-5.72315	5.43094	19.50642
H	-5.49225	3.65600	19.61767
C	-3.19415	2.16441	18.61299
H	-3.55192	1.74882	17.64178
H	-3.98446	1.96263	19.36752
H	-2.29502	1.58456	18.90548
C	1.73230	7.82750	23.46679
C	0.87022	7.98187	24.62112
C	-0.21109	8.86413	24.25360
C	-0.03024	9.24470	22.87191
C	1.16902	8.60677	22.38264
C	3.04595	7.09927	23.45936
H	3.83702	7.74113	23.91421
H	3.38092	6.83739	22.43508
H	2.99162	6.16047	24.04718
C	1.14084	7.40548	25.98516
H	1.89299	8.02402	26.52904
H	1.53963	6.37097	25.91925
H	0.22433	7.37916	26.61266
C	-1.28840	9.36794	25.17355
H	-0.96569	10.29825	25.69798
H	-1.54804	8.62588	25.95871
H	-2.22076	9.61099	24.62059
C	-0.88182	10.21513	22.09975
H	-0.51799	11.26126	22.23363

H	-1.94323	10.19449	22.42794
H	-0.86301	10.00337	21.00944
C	1.79350	8.81633	21.02970
H	2.46979	9.70306	21.04906
H	1.03038	9.00275	20.24410
H	2.40204	7.94730	20.70658
H	-4.80161	8.72940	26.21700
H	-7.22070	5.80118	25.14157
C	-6.34529	8.48846	22.01095
C	-5.60151	9.35441	22.89554
C	-5.71545	8.82639	24.23366
C	-6.52793	7.63477	24.17444
C	-6.62361	8.75773	20.55736
C	-4.95284	10.65349	22.49859
C	-5.21407	9.47019	25.49874
C	-7.03803	6.87262	25.36695
H	-7.52132	9.41096	20.44702
H	-6.83156	7.82659	19.98966
H	-5.78087	9.27867	20.05668
H	-5.70440	11.47707	22.46709
H	-4.48682	10.60056	21.49168
H	-4.16367	10.96264	23.21533
H	-6.04134	10.00512	26.02120
H	-4.41921	10.21776	25.29886
H	-8.00851	7.29977	25.71404
H	-6.33745	6.92365	26.22639
C	-6.91361	7.42226	22.80012
C	-7.84910	6.35384	22.30041
H	-8.90717	6.70475	22.33422
H	-7.63423	6.06879	21.24854
H	-7.79219	5.42965	22.91362

Vibrational frequencies (first 50 modes)

$vibrational spectrum

# mode	symmetry	wave number	IR intensity	selection rules	
#		cm**(-1)	km/mol	IR	RAMAN
1		0.00	0.00000	-	-
2		0.00	0.00000	-	-
3		0.00	0.00000	-	-
4		0.00	0.00000	-	-
5		0.00	0.00000	-	-
6		0.00	0.00000	-	-
7	a	5.84	0.00260	YES	YES
8	a	13.74	0.05627	YES	YES
9	a	15.66	0.11982	YES	YES
10	a	19.45	0.57683	YES	YES
11	a	21.25	0.17560	YES	YES
12	a	22.57	0.09953	YES	YES
13	a	23.31	0.12864	YES	YES
14	a	25.97	0.29225	YES	YES
15	a	27.46	0.22668	YES	YES
16	a	30.75	0.12825	YES	YES
17	a	31.49	0.16741	YES	YES
18	a	32.03	0.24333	YES	YES
19	a	33.28	0.23668	YES	YES
20	a	35.75	0.25612	YES	YES
21	a	37.17	0.21630	YES	YES
22	a	38.57	0.09600	YES	YES
23	a	39.15	0.02433	YES	YES
24	a	42.72	0.72238	YES	YES

25	a	44.15	0.53977	YES	YES
26	a	45.26	0.53185	YES	YES
27	a	49.35	0.72730	YES	YES
28	a	50.94	0.06713	YES	YES
29	a	52.75	1.80221	YES	YES
30	a	54.29	1.50157	YES	YES
31	a	55.64	0.83709	YES	YES
32	a	58.82	0.46098	YES	YES
33	a	59.36	0.12604	YES	YES
34	a	60.71	0.28631	YES	YES
35	a	62.37	0.52486	YES	YES
36	a	64.61	1.04904	YES	YES
37	a	67.78	0.58061	YES	YES
38	a	71.21	0.45835	YES	YES
39	a	72.63	0.22048	YES	YES
40	a	75.82	0.35691	YES	YES
41	a	78.90	0.51669	YES	YES
42	a	81.05	1.25317	YES	YES
43	a	84.76	3.27871	YES	YES
44	a	86.06	0.38418	YES	YES
45	a	90.11	0.95487	YES	YES
46	a	92.52	0.28345	YES	YES
47	a	94.79	0.03673	YES	YES
48	a	96.12	0.21384	YES	YES
49	a	96.94	0.17581	YES	YES
50	a	100.07	0.15112	YES	YES

5. References

[1] a) P. Csaszar, P. Pulay, *J. Mol. Struct.* **1984**, *114*, 31-34; b) R. Ahlrichs, M. Bar, M. Haser, H. Horn, C. Kolmel, *Chem. Phys. Lett.* **1989**, *162*, 165-169; c) K. Eichkorn, O. Treutler, H. Ohm, M. Haser, R. Ahlrichs, *Chem. Phys. Lett.* **1995**, *240*, 283-289; d) O. Treutler, R. Ahlrichs, *J. Chem. Phys.* **1995**, *102*, 346-354; e) K. Eichkorn, F. Weigend, O. Treutler, R. Ahlrichs, *Theor. Chem. Acc.* **1997**, *97*, 119-124; f) M. von Arnim, R. Ahlrichs, *J. Chem. Phys.* **1999**, *111*, 9183-9190; g) P. Deglmann, F. Furche, *J. Chem. Phys.* **2002**, *117*, 9535-9538; h) P. Deglmann, F. Furche, R. Ahlrichs, *Chem. Phys. Lett.* **2002**, *362*, 511-518; i) P. Deglmann, K. May, F. Furche, R. Ahlrichs, *Chem. Phys. Lett.* **2004**, *384*, 103-107; j) A. Baldes, F. Weigend, *Mol. Phys.* **2013**, *111*, 2617-2624; k) F. Furche, R. Ahlrichs, C. Hattig, W. Klopper, M. Sierka, F. Weigend, *WIREs Comput. Mol. Sci.* **2014**, *4*, 91-100.

[2] M. J. Frisch, G. W. Trucks, H. B. Schlegel, G. E. Scuseria, M. A. Robb, J. R. Cheeseman, G. Scalmani, V. Barone, B. Mennucci, G. A. Petersson, H. Nakatsuji, M. Caricato, X. Li, H. P. Hratchian, A. F. Izmaylov, J. Bloino, G. Zheng, J. L. Sonnenberg, M. Hada, M. Ehara, K. Toyota, R. Fukuda, J. Hasegawa, M. Ishida, T. Nakajima, Y. Honda, O. Kitao, H. Nakai, T. Vreven, J. J. A. Montgomery, J. E. Peralta, F. Ogliaro, M. Bearpark, J. J. Heyd, E. Brothers, K. N. Kudin, V. N. Staroverov, T. Keith, R. Kobayashi, J. Normand, K. Raghavachari, A. Rendell, J. C. Burant, S. S. Iyengar, J. Tomasi, M. Cossi, N. Rega, J. M. Millam, M. Klene, J. E. Knox, J. B. Cross, V. Bakken, C. Adamo, J. Jaramillo, R. Gomperts, R. E. Stratmann, O. Yazyev, A. J. Austin, R. Cammi, C. Pomelli, J. W. Ochterski, R. L. Martin, K. Morokuma, V. G. Zakrzewski, G. A. Voth, P. Salvador, J. J. Dannenberg, S. Dapprich, A. D. Daniels, O. Farkas, J. B. Foresman, J. V. Ortiz, J. Cioslowski, D. J. Fox, Gaussian, Inc., Wallingford CT, **2013**.

[3] E. D. Glendening, J. K. Badenhoop, A. E. Reed, J. E. Carpenter, J. A. Bohmann, C. M. Morales, P. Karafiloglou, C. R. Landis, F. Weinhold, Theoretical Chemistry Institute, University of Wisconsin, Madison, WI, **2018**.

[4] M. Suenaga, *J. Comput. Chem Jpn.* **2005**, *4*, 25-32.

[5] http://jmol.sourceforge.net/ ed.
